# Depression in South Korean Adolescents Captured by Text and Opinion Mining of Social Big Data

**DOI:** 10.3390/ijerph20176665

**Published:** 2023-08-28

**Authors:** Juyoung Song, Tae-Min Song, Sangho Lee, Dong-Chul Seo

**Affiliations:** 1 Administration of Justice, Pennsylvania State University, Schuylkill, PA 17972, USA; juyoung81@gmail.com; 2 Gachon University Graduate School of Industry & Environment, Seoul 13120, Republic of Korea; tmsong01@hanmail.net; 3 HealthMax Co., Ltd., Seoul 06078, Republic of Korea; lsh@healthmax.co.kr; 4 Department of Applied Health Science, Indiana University School of Public Health, Bloomington, IN 47405, USA

**Keywords:** adolescents, text mining, emotional susceptibility, depression, social big data

## Abstract

Depression in adolescence is recognized as an important social and public health issue that interferes with continued physical growth and increases the likelihood of other mental disorders. The goal of this study was to examine online documents posted by South Korean adolescents for 3 years through the text and opinion mining of collectable documents in order to capture their depression. The sample for this study was online text-based individual documents that contained depression-related words among adolescents, and these were collected from 215 social media websites in South Korea from 1 January 2012 to 31 December 2014. A sentiment lexicon was developed for adolescent depressive symptoms, and such sentiments were analyzed through opinion mining. The depressive symptoms in the present study were classified into nine categories as suggested by the Diagnostic and Statistical Manual for Mental Disorders, 5th Edition (DSM-5). The association analysis and decision tree analysis of data mining were used to build an efficient prediction model of adolescent depression. Opinion mining indicated that 15.5% were emotionally stable, 58.6% moderately stressed, and 25.9% highly distressed. Data mining revealed that the presence of depressed mood most of the day or nearly every day had the greatest effect on adolescents’ depression. Social big data analysis may serve as a viable option for developing a timely response system for emotionally susceptible adolescents. The present study represents one of the first attempts to investigate depression in South Korean adolescents using text and opinion mining from three years of online documents that originally amounted to approximately 3.1 billion documents.

## 1. Introduction

Even though depression is a familiar part of the vocabulary for men and women of all ages and is referred to as “modern people’s mental cold” in psychopathology, its harmful effects are serious [1]. Depression in adolescence is recognized as an important social and public health issue that interferes with continued physical growth and increases the likelihood of other mental disorders. It usually takes chronic and recurrent courses and often bears an undesirable influence on interpersonal functioning and academic achievements [1]. Depression is a social issue because it is associated with suicidal thoughts and attempts [2,3]. Indeed, many people who commit suicide turn out to have suffered from depression [2,4]. In addition, thoughts of self-harm are considered suicide risk factors and are also highly related to mental illnesses such as major depressive disorder [2,5].

Most people consider depression a “private matter”, and because they do not want to be called a mentally ill patient, they tend to refuse treatments [6,7]. The number of patients with depression is about 350 million worldwide [8]. By using a Korean female adolescent sample, the study suggests that depressive symptoms partially mediate the relationship between ADHD symptoms and suicidal ideation [9]. However, more than 50% of these patients are not utilizing appropriate therapies or medical care [10]. The prevalence of depressive disorders in adults 19 years or older in South Korea was 6.7% in 2014, and only 18.2% of the affected patients sought counseling or treatment for mental problems [11]. Even though the rate of adolescents experiencing depressive mood in South Korea has been overall decreasing from 32.8% in 2011, 30.5% in 2012, 30.9% in 2013, to 26.7% in 2014, the rate is still high [11].

Prior research has shown that a number of sociodemographic, lifestyle, and disease-related factors affect depression. For example, being female [12,13], low social capital in the family [14,15], and experience of divorce or separation [16] are related to a higher likelihood of depression. Meanwhile, as the influence of social networking sites (SNS hereafter) increases, SNS are becoming not only a communication tool for ordinary daily matters or chats but also a space where one can express or listen to depressive feelings, stress, or worries [17].

Especially given that almost all Korean adolescents are using SNS on a daily basis, Korean adolescents who have depressive symptoms or mood are highly likely to leave postings on online media such as Twitter, blogs, cafés, and bulletin boards. The emotional contagion theory states that whatever emotion is expressed by someone, such emotion tends to be transferred similarly to another person who is exposed to it [18]. Morris et al. and Kramer, Guillory and Hancock affirm this, reporting that buzzes posted on Facebook affect the emotions of other users who read them [19,20]. Krasnova, Wenninger, Widjaja and Buxmann noted “Facebook Envy”, which refers to the phenomenon that Facebook postings stir people’s feelings of envy as some use Facebook as an instrument to show off their happy lives rather than as a communication space [21]. Other researchers reported a similar finding that long-term use of Facebook may lead people to feel depressed [22,23]. The American Academy of Pediatrics stated that adolescents’ exposure to social media such as Facebook could help adolescents feel depressed [24]. Lin et al. surveyed 1787 adults ages 19 to 32 about social media use and depression and found that social media use was significantly associated with increased depression [25].

Regardless of the similar or dissimilar feelings that SNS postings stimulate, SNS are increasingly becoming a space where one can express or be exposed to stressful feelings or worries as well as cheerful or pleasant emotions and stories. Thus, it is plausible that an analysis of online postings among Korean adolescents may shed light on the characteristics and patterns of such postings and help identify adolescents who are emotionally susceptible. Better yet, such an analysis could help establish an automatic online monitoring system (e.g., artificial intelligence) that could monitor and capture online postings of emotionally susceptible adolescents and provide them with ballooned texts of helpful information on a real-time basis. Although a prior study was conducted to analyze depression-related Twitter postings, it was based on relatively small amounts of tweets during only a 2-month period [26].

The purpose of this study is to analyze the characteristics and forms of depression in adolescents and to predict risk factors through social big data analysis. Further, this research intends to demonstrate whether social big data analysis may serve well in depicting the probability of depression in adolescents using social media on a regular basis. It may also be an important guide for the development of an online response system for emotionally susceptible adolescents. The present study analyzed online documents posted by South Korean adolescents that contained depression-related words for 3 years from 1 January 2012 to 31 December 2014 through the text and opinion mining of collectable documents in order to capture their depression. The study conducted text mining and opinion mining of online text-based documents in online channels, which allowed for the approximate identification of adolescents who were emotionally susceptible. The study hypothesized that the nine depressive symptoms suggested by the Diagnostic and Statistical Manual for Mental Disorders, 5th Edition (DSM-5), have differential predictability of depression.

## 2. Materials and Methods

### 2.1. Sample

**Figure 1:**
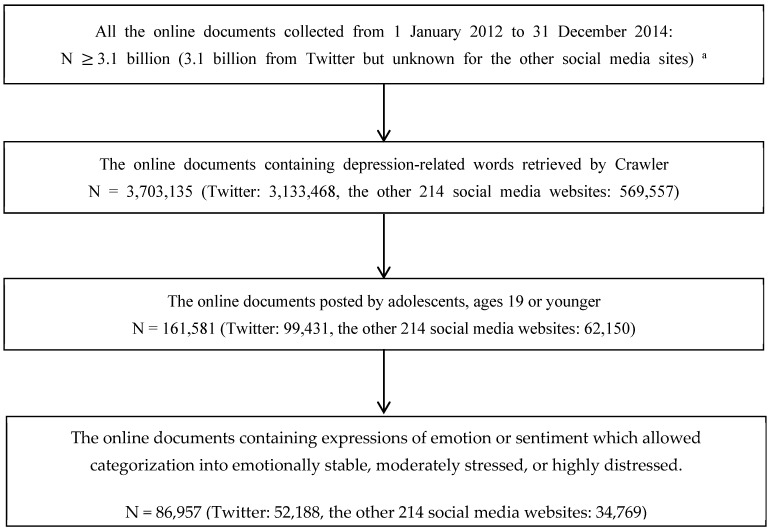
Flowchart of sample selection. Note. ^a^ Whereas the entire number of online posts during the study period was available for Twitter due to the full crawler retrieval method, it was not available for the other social media websites due to the focused crawler retrieval method.

The procedures for the collection and classification of online documents related to adolescent emotional susceptibility (i.e., depression-related words) in the present study are illustrated in Figure 1. The sample for this study was online text-based individual documents that contained depression-related words among adolescents, and these were collected from 215 popular social media websites in South Korea (i.e., Twitter, 199 online news sites, 9 online bulletin boards, 4 blogs, and 2 online cafés) from 1 January 2012 to 31 December 2014. Twitter crawling uses the Twitter API (application programming interface) to crawl. To use the Twitter API, register an application on the Twitter development site and access and collect public information.

As shown in Figure 1, out of more than 3.10 billion posts from the 3 years, a total of 3,703,135 contained depression-related words. Noise was minimized by excluding documents that contained predetermined stop-words. In order to limit the study sample to adolescents, only documents that contained such descriptors as adolescents, secondary school students, or ages 19 or younger were selected and included in the sample, which reduced the sample to 161,581. Among the documents from these adolescents, 86,957 documents contained expressions of emotion or sentiment which allowed categorization into emotionally stable, moderately stressed, or highly distressed.

### 2.2. Measures

For the purpose of collecting and analyzing social big data related to adolescent depression, we constructed an adolescent depression management ontology on the basis of Ontology Development 101 [27] and developed a theme classification system and a terminology system for youth depression management [28]. For the natural language processing of the collected data, morpheme analysis was conducted using methodologies such as head–tail classification, left–right and right–left analysis, and syllable unit analysis. The data refining was performed using keyword extraction through morpheme analysis of the collected documents, and the documents relating to advertisement posts were filtered and excluded.

#### 2.2.1. Dependent Variable

The dependent variable of the present study was depression. Once all of the relevant online text documents were retrieved, composing elements (such as nouns, verbs, adjectives, prepositions, and postpositional words) were identified and classified based on analysis of linguistic morphology of texts through text mining. Then, opinion mining was performed using the ontology developed for analyzing adolescents’ depressive mood, and one of three states of depression (i.e., emotionally stable, moderately stressed, and highly distressed) was assigned to each document. The emotionally stable state (coded 1) applies to documents that mention having no/little stress, no/little depression, or having a sense of happiness or peace. The highly distressed state (coded 3) refers applies to documents that mention having serious stress, worries, fear, insomnia, depression, suicidal thoughts, or victimization to school violence. The moderately stressed state (coded 2) applies to documents that mention things other than codes 1 and 3.

#### 2.2.2. Independent Variables

The major independent variable was the nine binary (yes/no) variables of depressive symptoms suggested by the DSM-5. Specifically, DSM1 (words included in the documents: depressed, depressed feeling, depression tweet, unhappiness, sorrow, cry, deep sadness, grief, gloom, depressive disorder, depression, neurotic depression, psychotic depression, severe depression, and chronic depression) refers to depressed mood most of the day or nearly every day; DSM2 (words included in the documents: psychotic depression, character depression, psychotic depression, mild depression, hypothermia, emotional abnormality, powerless, and asthenia) markedly diminished interest or pleasure in all or almost all activities most of the day or nearly every day; DSM3 (words included in the documents: loss of appetite, weight change, weight increase, weight decrease, fat, obese, corpulent, fleshy, and increased appetite) a significant weight loss or weight gain, or decrease/increase in appetite nearly every day; DSM4 (words included in the documents: dyssomnia, lethargy, sleeping pills, drowsiness, sleep, lack of sleep, excessive sleep, narcolepsy, insomnia, and sleep disorders) insomnia or hypersomnia nearly every day; DSM5 (words included in the documents: hypomania, amnesia, psychiatric symptoms, auditory hallucination, delusion, mental symptoms, insanity, anxiety, jitter, phobia, irritancy, agitation, excitation, psychomotor agitation, nerve-racking, impulsiveness, impulsion, worry, incomplete, psychomotor retardation, anxiety disorder, neurosis, phobia, panic disorder, generalized anxiety disorder, and panic disorder) psychomotor agitation or retardation nearly every day; DSM6 (words included in the documents: fatigue, feeling confused, confusion, chaos, lightheaded, dizziness, inertia, and weariness) fatigue or loss of energy nearly every day; DSM7 (words included in the documents: worthlessness, feeling of guilt, inappropriate guilt, sense of sin, sense of sins, guilty conscience, guilt, remorse, and blame oneself) feelings of worthlessness or excessive or inappropriate guilt; DSM8 (words included in the documents: dependence, dependent, dependent personality, obedient personality, submissive, obedience, decline of concentration, indecisiveness, decline of brain activity, brain, brain activity, decline of brain function, brain function, ADHD, and attention deficit hyperactivity disorder) diminished ability to think or concentrate, or indecisiveness nearly every day; and DSM9 (words included in the documents: suicidal tendency, suicide, suicidal impulse, suicidal ideation, and suicide-related behaviors) recurrent thoughts of death, recurrent suicidal ideation without a specific plan, or a suicide attempt or a specific plan for committing suicide. For statistical analysis, each expression that contained each of the depression symptoms was coded as 1 versus 0.

### 2.3. Data Analysis

Online documents related to online adolescent depression were collected. After extracting keywords through morphological analysis, risk factors related to adolescent depression were organized by classifying them into nine depressive symptoms suggested by DSM-5.

In the present study, association analysis of data mining and decision tree analysis, which does not require statistical assumptions, were used to build an efficient prediction model, so that adolescents’ depressive symptoms mentioned in online channels could be used to identify depression. For the association analysis, the Apriori principle algorithm proposed by Agrawal and Srikant was used [29]. The Apriori algorithm identifies associations between two or more words included in an online document or transaction. Association rules are predicated on “support” (used to remove rules that appear less frequently) and “confidence” (used to gauge the strength of the association between words). Association analysis involves the generation of frequent item sets that satisfy a minimal support criterion as defined by the researcher. Of these item sets, those that satisfy a minimal confidence criterion and a lift of at least 1 are selected [26]. Evaluation of the association analysis used to predict adolescents’ depression was carried out using the criteria of support 0.001 and confidence 0.1. The analysis algorithm used to form the decision tree in the present study was Chi-squared automatic interaction detection [30]. As a stopping rule, the minimum number of cases for the parent node was set at 100 and for the child node at 50, while the depth was set at 3 [30]. IBM SPSS version 24.0 was used for decision tree analysis and R version 3.5.0 was used for association analysis. In regard to ethical consideration of the research, this study was conducted after obtaining approval from Institutional Review Board of the Korea Institute for Health and Social Affairs (NO. 2014-23). The research used social big data collected by the Korea Institute for Health and Social Affairs. The social big data collected did not include personal identification information, thereby ensuring the anonymity and confidentiality of the subjects.

## 3. Results

As shown in Table 1, opinion mining of online documents (buzzes) indicated that 15.5% of subjects were emotionally stable, 58.6% were moderately stressed, and 25.9% were highly distressed (25.2% in 2012, 27.9% in 2013, and 24.5% in 2014). Of the nine DSM-5 symptoms, “feeling depressed most of the day or nearly every day” appeared the most frequently (42.4%), followed by “feeling anxiety” (15.9%) and “having recurrent thoughts of death” (12.8%).

As shown in Table 2, of all of the buzzes that showed two or more depressive symptoms, 44.2% were associated with a highly distressed status. In terms of online channels, more emotionally stable buzzes were found in blogs (38.1%) than in other places (29.7% in online cafés, 24.3% in online news, 16.9% in online boards, and 6.5% on Twitter).

**Figure 2:**
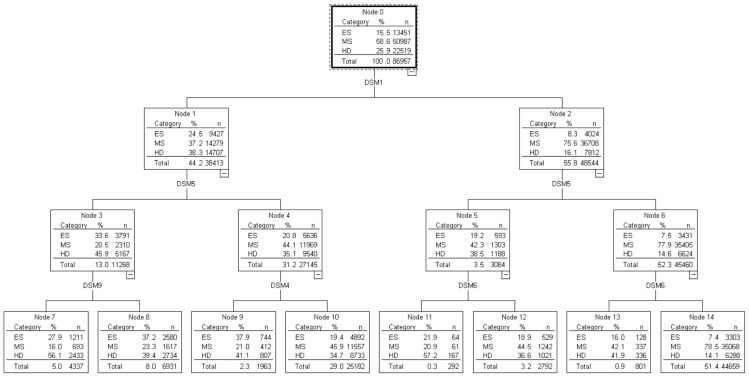
Decision Tree Analysis of Emotional Susceptibility.

The result of decision tree analysis of depression is shown in Figure 2 and a corresponding profit chart is shown in Table 3. The presence of DSM1 (depressed mood most of the day or nearly every day) was found to have the greatest effect on depression. If DSM1 symptoms were present, the likelihood of finding highly distressed individuals increased from 25.9% to 38.3% and that of finding moderately stressed individuals decreased from 58.6% to 37.2%. If both the DSM1 and DSM5 (psychomotor agitation or retardation nearly every day) symptoms were present, the likelihood of finding highly distressed individuals increased from 38.3% to 45.9% and that of finding moderately stressed individuals decreased from 37.2% to 20.5%. If all three symptoms of DSM1, DSM5, and DSM9 (recurrent thoughts of death, recurrent suicidal ideation without a specific plan, or a suicide attempt or a specific plan for committing suicide) were present, then the likelihood of finding highly distressed individuals increased from 45.9% to 51.6% and that of finding moderately stressed individuals decreased from 20.5% to 16.0%.

As shown in Table S1 (Supplementary Materials), the association analysis of depression indicates that highest level of confidence (0.675) was associated with a combination of six variables (DSM2, DSM3, DSM4, DSM6, DSM7, and DSM8) and a highly distressed emotional state with an increase of 2.605. This means that when the six depressive symptoms are mentioned in online documents, the level of confidence of finding a highly distressed emotional state is about 67.5% and the risk for feeling highly distressed is about 2.605 times the risk when these six symptoms are not mentioned.

## 4. Discussion

### 4.1. Contributions

The present study is one of the first attempts to investigate the depression of South Korean adolescents using text and opinion mining from three years of online documents that originally amounted to approximate 3.1 billion documents. One of the major hypotheses that drove this investigation was that the text and opinion mining of collectable documents in online channels would allow for the approximate identification of adolescents who are emotionally susceptible. The results of the present study showed that 25.2% in 2012, 27.9% in 2013, and 24.5% in 2014 of the adolescents were highly distressed. These numbers are compared to 30.5% in 2012, 30.9% in 2013, and 26.7% in 2014 of surveyed Korean adolescents who reported feeling sad or depressed in the past two weeks in a national online surveillance among 75,000 students [11]. Although the percentage differences are statistically significant between the national surveillance data and the data from the present study, one could argue that the differences are not large. It appears that there is potential for the text and opinion mining of online documents to identify adolescents who are emotionally susceptible.

The differential association between depression and the nine symptoms of the Diagnostic and Statistical Manual for Mental Disorders, 5th Edition, deserves mention. In the present study, emotionally susceptible adolescents were associated with depressive symptoms in the order of DSM1 (depressed mood most of the day or nearly every day), DSM5 (psychomotor agitation or retardation nearly every day), and DSM9 (recurrent thoughts of death, suicide attempts or a specific plan for committing suicide). This result contrasts with that of Cavazos–Rehg et al. who analyzed Twitter postings and found depressive symptoms to the order of DSM1, DSM7 (feelings of worthlessness or excessive guilt), and DSM9 [31]. For both studies, DSM1 and DSM9 were mentioned as important predictors for emotionally susceptible adolescents. This implies that these two symptoms found in online documents could serve as major diagnostic criteria for depressive symptoms in adolescents.

Measurement of the depression of adolescents should be approached with clinical methods. It is difficult to measure the entire population of adolescents due to the lack of population-based-data. The national surveillance system survey is the most common tool used to measure depression in adolescents. The survey has a high possibility of error due to social stigma and social desirability bias. Adolescents commonly express their feelings, depression, and stress in daily life on social network services. The information related to depression in adolescents appearing online can be collected with less bias than the information related to depression measured in the national surveillance system survey. Therefore, analyzing online documents related to emotional expressions or psychological crisis behaviors of adolescents could be a more effective response to the problems of youth exposed to the risk of depression as well as the prediction of risk signs.

The findings of the present study have policy implications for preventing and responding to depression risk in South Korean adolescents. Given that adolescents express or are exposed to stressful feelings, depressive mood, or even suicidal ideation online, and that such emotion or ideation can be transferred to another person who is exposed to it, it may be necessary to establish an online depression monitoring system. Further, for a timely response to adolescents at risk in cyber space, it would be desirable to build a system that captures the online postings of emotionally susceptible adolescents and provides them with ballooned texts of helpful information on a real-time basis would be needed.

### 4.2. Limitations

The study has limitations. First, caution is warranted to guard interpretations against ecological fallacy. Given that the present study did not perform analysis on individual characteristics but used the data of the entire group of individual members, an ecological fallacy could occur if the results are applied to individuals [17]. Second, if some of the analyzed online documents (buzzes) that appeared to be written by adolescents were actually posted by adults that used adolescent keywords (e.g., under the age of 19, elementary school students, middle school students, and high school students), the results might have been confounded. Third, this research assumed that individuals did not engage in disinformation in their postings. Even if some individuals posted false information in order to mislead others, the impact on the study findings might be minimal as such individuals, if any, would comprise only a minuscule fraction of the sample given the topic. Despite these limitations, the present study contributes to the literature and the field by suggesting a new analytical method that would allow for building a timely response system to provide assistance to emotionally susceptible adolescents.

## 5. Conclusions

The present study is one of the first attempts to investigate depression in South Korean adolescents using text and opinion mining from three years of online documents that originally amounted to approximate 3.1 billion documents. It appears that there is potential for the text and opinion mining of online documents to identify adolescents who are emotionally susceptible. The differential association between depression and the nine symptoms of the Diagnostic and Statistical Manual for Mental Disorders, 5th Edition, deserves mention. In the present study, emotionally susceptible adolescents were associated with depressive symptoms to the order of DSM1, namely depressed mood most of the day or nearly every day; DSM5, namely psychomotor agitation or retardation nearly every day; and DSM9, namely recurrent thoughts of death, suicide attempts, or a specific plan for committing suicide. It may be necessary to establish an online depression monitoring system and a timely response to adolescents at risk in cyber space to prevent or help adolescents’ depression risks.

## Figures and Tables

**Table 1:** Distribution of Emotional Susceptibility and Depressive Symptoms.

Emotional Susceptibility		N	(%)	Depressive Symptom ^1^	N	(%)
2012	Emotionally stable	4294	(16.8)	DSM1	38,413	(42.4)
	Moderately stressed	14,794	(57.9)	DSM2	4886	(5.4)
	Highly distressed	6441	(25.2)	DSM3	2227	(2.5)
2013	Emotionally stable	4694	(15.9)	DSM4	5971	(6.6)
	Moderately stressed	16,527	(56.1)	DSM5	14,352	(15.9)
	Highly distressed	8232	(27.9)	DSM6	6508	(7.2)
2014	Emotionally stable	4463	(14.0)	DSM7	3127	(3.5)
	Moderately stressed	19,666	(61.5)	DSM8	3429	(3.8)
	Highly distressed	7846	(24.5)	DSM9	11,624	(12.8)
Total	Emotionally stable	13,451	(15.5)	Total	90,535	
	Moderately stressed	50,987	(58.6)			
	Highly distressed	22,519	(25.9)			
	Total	86,957				

Note. DSM1: depressed mood most of the day, nearly every day, as indicated by subjective report; DSM2: markedly diminished interest or pleasure in all, or almost all, activities most of the day, nearly every day; DSM3: significant weight loss when not dieting or weight gain, or decrease or increase in appetite nearly every day; DSM4: insomnia or hypersomnia nearly every day; DSM5: psychomotor agitation or retardation nearly every day; DSM6: fatigue or loss of energy nearly every day; DSM7: feelings of worthlessness or excessive or inappropriate guilt; DSM8: diminished ability to think or concentrate, or indecisiveness, nearly every day; DSM9: recurrent thoughts of death, recurrent suicidal ideation without a specific plan, or a suicide attempt or a specific plan for committing suicide. ^1^ Depressive symptoms may be counted multiple times.

**Table 2:** Crosstabulation of Emotional Susceptibility with Depressive Symptoms and Online Channels.

Characteristics		Emotionally Stable		Moderately Stressed		Highly Distressed		*χ*^2^ (*p*)
Depressive Symptoms	1	3998	(16.8)	12,259	(51.5)	7524	(31.6)	3334.1 (<0.001)
	≥2	6448	(30.5)	5352	(25.3)	9336	(44.2)	
Online Channels	Twitter	3369	(6.5)	40,634	(77.9)	8185	(15.7)	22,354.9 (<0.001)
	Blog	4586	(38.1)	3096	(31.4)	4352	(36.2)	
	Cafe	2500	(29.7)	2650	(31.4)	3277	(38.9)	
	Board	1084	(16.9)	1850	(28.8)	3494	(54.4)	
	News	1912	(24.3)	2757	(35.0)	3211	(40.7)	

Note. Numbers in parentheses are percentages across rows.

**Table 3:** Profit Chart in Modeling Depression.

Classification	Node	Profit Index				Cumulative Index			
		*n*	%	Profit (%)	Index (%)	*n*	%	Profit (%)	Index (%)
Highly Distressed	11	292	0.3	0.7	220.8	292	0.3	0.7	220.8
	7	4337	5.0	10.8	216.6	4629	5.3	11.5	216.9
	13	801	0.9	1.5	162.0	5430	6.2	13.0	208.8
	9	1963	2.3	3.6	158.7	7393	8.5	16.6	195.5
	8	6931	8.0	12.1	152.3	14,324	16.5	28.8	174.6
	12	2792	3.2	4.5	141.2	17,116	19.7	33.3	169.2
	10	25,182	29.0	38.8	133.9	42,298	48.6	72.1	148.2
	14	44,659	51.4	27.9	54.4	86,957	100	100.0	100.0
Moderately Stressed	14	44,659	51.4	68.8	133.9	44,659	51.4	68.8	133.9
	10	25,182	29.0	22.7	78.3	69,841	80.3	91.4	113.9
	12	2792	3.2	2.4	75.9	72,633	83.5	93.9	112.4
	13	801	0.9	0.7	71.8	73,434	84.4	94.5	112.0
	8	6931	8.0	3.2	39.8	80,365	92.4	97.7	105.7
	9	1963	2.3	0.8	35.8	82,328	94.7	98.5	104.1
	11	292	0.3	0.1	35.6	82,620	95.0	98.6	103.8
	7	4337	5.0	1.4	27.3	86,957	100.0	100.0	100.0
Emotionally Stable	9	1963	2.3	5.5	245	1963	2.3	5.5	245.0
	8	6931	8.0	19.2	240.6	8894	10.2	24.7	241.6
	7	4337	5.0	9.0	180.5	13,231	15.2	33.7	221.6
	11	292	0.3	0.5	141.7	13,523	15.6	34.2	219.9
	10	25,182	29	36.4	125.6	38,705	44.5	70.6	158.5
	12	2792	3.2	3.9	122.5	41,497	47.7	74.5	156.1
	13	801	0.9	1.0	103.3	42,298	48.6	75.4	155.1
	14	44,659	51.4	24.6	47.8	86,957	100.0	100.0	100.0

Note. Node numbers refer to the nodes in Figure 2.

## Data Availability

The data can be provided upon request to the Korea Institute for Health and Social Affairs and signing on the data use agreement.

## Supplementary Materials

The following supporting information can be downloaded at: https://www.mdpi.com/article/10.3390/ijerph20176665/s1, Table S1: Association Rules of Depressive Symptoms.
