# Supplementary material for: Depression in South Korean Adolescents Captured by Text and Opinion Mining of Social Big Data
Source: Int J Environ Res Public Health. 2023 Aug 28;20(17):6665. doi: 10.3390/ijerph20176665 (PMC10487740; doi:10.3390/ijerph20176665)
Supplement: Supplementary file 1 [file ijerph-20-06665-s001.zip › ijerph-2535752-supplementary materials.pdf]

Table S1. Association Rules of Depressive Symptoms

	Rule	Support	Confidence	Lift
Highly Distressed	{DSM2,DSM3,DSM4,DSM6,DSM7,DSM8} → { highly distressed }	0.0022424	0.6747405	2.605507
	{DSM1,DSM2,DSM3,DSM4,DSM6,DSM7,DSM8} → { highly distressed }	0.0022424	0.6747405	2.605507
	{DSM2,DSM3,DSM6,DSM7,DSM8} → { highly distressed }	0.0023344	0.6744186	2.604264
	{DSM1,DSM2,DSM3,DSM6,DSM7,DSM8} → { highly distressed }	0.0023344	0.6744186	2.604264
	{DSM3,DSM6,DSM7,DSM8} → { highly distressed }	0.0024494	0.6719243	2.594632
	{DSM1,DSM3,DSM6,DSM7,DSM8} → { highly distressed }	0.0024494	0.6719243	2.594632
	{DSM2,DSM3,DSM4,DSM6,DSM7,DSM8,DSM9,} → { highly distressed }	0.0021389	0.6714801	2.592917
	{DSM1,DSM2,DSM3,DSM4,DSM6,DSM7,DSM8,DSM9} → { highly distressed }	0.0021389	0.6714801	2.592917
	{DSM2,DSM3,DSM6,DSM7,DSM8,DSM9} → { highly distressed }	0.0022309	0.6712803	2.592145
	{DSM1,DSM2,DSM3,DSM6,DSM7,DSM8,DSM9} → { highly distressed }	0.0022309	0.6712803	2.592145
Moderately Stressed	{DSM1} → { moderately stressed }	0.1642075	0.3717231	0.633964
	{DSM9} → { moderately stressed }	0.0434927	0.3253613	0.554895
	{DSM1,DSM9} → { moderately stressed }	0.0282438	0.2604730	0.444229
	{DSM5} → { moderately stressed }	0.0415492	0.2517419	0.429339
	{DSM2} → { moderately stressed }	0.0115919	0.2063037	0.351845
	{DSM1,DSM5} → { moderately stressed }	0.0265648	0.2050053	0.349631
	{DSM6} → { moderately stressed }	0.0150879	0.2015980	0.343820
	{DSM8} → { moderately stressed }	0.0076474	0.1939341	0.330749
	{DSM7} → { moderately stressed }	0.0068539	0.1905980	0.325060
	{DSM1,DSM8} → { moderately stressed }	0.0069229	0.1867246	0.318453
Emotionally Stable	{DSM1,DSM4,DSM8} → { emotionally stable }	0.0073829	0.4032663	2.607005
	{DSM4,DSM8} → { emotionally stable }	0.0074749	0.4000000	2.585890
	{DSM6,DSM8} → { emotionally stable }	0.0060604	0.3944611	2.550082
	{DSM4,DSM6,DSM8} → { emotionally stable }	0.0044619	0.3943089	2.549098
	{DSM1,DSM4,DSM6,DSM8} → { emotionally stable }	0.0044274	0.3940635	2.547511
	{DSM1,DSM4,DSM6 → { emotionally stable }	0.0120404	0.3940534	2.547447
	{DSM1,DSM6,DSM8} → { emotionally stable }	0.0058879	0.3914373	2.530534
	{DSM4,DSM6} → { emotionally stable }	0.0122589	0.3906193	2.525246
	{DSM1, DSM8 → { emotionally stable }	0.0144554	0.3898883	2.520520
	{DSM8} → { emotionally stable }	0.0152719	0.3872849	2.503690

Note. DSM1: depressed mood most of the day, nearly every day, as indicated by either subjective report; DSM2: markedly diminished interest or pleasure in all, or almost all, activities most of the day, nearly every day; DSM3: significant weight loss when not dieting or weight gain or decrease or increase in appetite nearly every day; DSM4: insomnia or hypersomnia nearly every day; DSM5: psychomotor agitation or retardation nearly every day; DSM6: fatigue or loss of energy nearly every day; DSM7: feelings of worthlessness or excessive or inappropriate guilt; DSM8: diminished ability to think or concentrate, or indecisiveness, nearly every day; DSM9: recurrent thoughts of death, recurrent suicidal ideation without a specific plan, or a suicide attempt or a specific plan for committing suicide.
